# Impacts of Multilayer Hybrid Coating on PSF Hollow Fiber Membrane for Enhanced Gas Separation

**DOI:** 10.3390/membranes10110335

**Published:** 2020-11-11

**Authors:** Rosyiela Azwa Roslan, Woei Jye Lau, Gwo Sung Lai, Abdul Karim Zulhairun, Yin Fong Yeong, Ahmad Fauzi Ismail, Takeshi Matsuura

**Affiliations:** 1 Advanced Membrane Technology Research Centre (AMTEC), Universiti Teknologi Malaysia, Skudai 81310, Malaysia; rosyielaazwa.roslan@yahoo.com (R.A.R.); gslai90@gmail.com (G.S.L.); zulhairun@petroleum.utm.my (A.K.Z.); afauzi@utm.my (A.F.I.); 2 School of Chemical and Energy Engineering, Universiti Teknologi Malaysia, Skudai 81310, Malaysia; 3 Department of Chemical Engineering, Universiti Teknologi PETRONAS, Bandar Seri Iskandar 32610, Malaysia; yinfong.yeong@utp.edu.my; 4 Department of Chemical Engineering, Industrial Membrane Research Institute, University of Ottawa, 161 Louis Pasteur Street, P.O. Box 450, Station A, Ottawa, ON K1N 6N5, Canada

**Keywords:** multilayer coating, nanomaterials, graphene oxide, membrane, gas separation

## Abstract

One of the most critical issues encountered by polymeric membranes for the gas separation process is the trade-off effect between gas permeability and selectivity. The aim of this work is to develop a simple yet effective coating technique to modify the surface properties of commonly used polysulfone (PSF) hollow fiber membranes to address the trade-off effect for CO_2_/CH_4_ and O_2_/N_2_ separation. In this study, multilayer coated PSF hollow fibers were fabricated by incorporating a graphene oxide (GO) nanosheet into the selective coating layer made of polyether block amide (Pebax). In order to prevent the penetration of Pebax coating solution into the membrane substrate, a gutter layer of polydimethylsiloxane (PDMS) was formed between the substrate and Pebax layer. The impacts of GO loadings (0.0–1.0 wt%) on the Pebax layer properties and the membrane performances were then investigated. XPS data clearly showed the existence of GO in the membrane selective layer, and the higher the amount of GO incorporated the greater the *sp2* hybridization state of carbon detected. In terms of coating layer morphology, increasing the GO amount only affected the membrane surface roughness without altering the entire coating layer thickness. Our findings indicated that the addition of 0.8 wt% GO into the Pebax coating layer could produce the best performing multilayer coated membrane, showing 56.1% and 20.9% enhancements in the CO_2_/CH_4_ and O_2_/N_2_ gas pair selectivities, respectively, in comparison to the membrane without GO incorporation. The improvement is due to the increased tortuous path in the selective layer, which created a higher resistance to the larger gas molecules (CH_4_ and N_2_) compared to the smaller gas molecules (CO_2_ and O_2_). The best performing membrane also demonstrated a lower degree of plasticization and a very stable performance over the entire 50-h operation, recording CO_2_/CH_4_ and O_2_/N_2_ gas pair selectivities of 52.57 (CO_2_ permeance: 28.08 GPU) and 8.05 (O_2_ permeance: 5.32 GPU), respectively.

## 1. Introduction

Natural gas is a naturally occurring hydrocarbon gas mixture containing mainly methane (CH_4_), carbon dioxide (CO_2_), nitrogen (N_2_), and higher hydrocarbons (C_n_H_2n+2_). The composition of natural gas, however, varies from one location to another, and its quality highly depends on the concentration of the contaminants [1]. In general, the removal of CO_2_ is essential before feeding the natural gas to a pipeline, as CO_2_ is highly corrosive and could destroy the pipelines and equipment [2]. In addition, concentrated/enriched gas such as oxygen (O_2_) is important for medical applications (for patients suffering from a low O_2_ level in their blood) and for indoor air quality improvement [3,4]. For these purposes, gas separation technology is required.

Conventionally, there are two main approaches that can be used to separate a gas mixture. There are ultra-low-temperature cryogenic distillation and non-cryogenic approaches based on pressure swing adsorption (PSA) [5,6,7]. However, membrane-based technology has received increasing attention over the past decade as it offers several main advantages over conventional techniques, including lower energy consumption, process simplicity, reduced space requirements, being free of chemical usage, and scalability [8].

Although polymeric membranes made of different commercial materials—e.g., polysulfone (PSF), polyethersulfone (PES), polyvinylidene fluoride (PVDF), polyamide (PA), polyimide (PI), and polybenzimidazole (PBI)—could be used for gas separation [9,10,11], most of them still suffer from the trade-off limitation between permeability and selectivity [12,13]. In order to address this problem, researchers always come up with new strategies to modify existing polymer-based membranes, aiming to overcome their limitations.

Developing membranes using novel high-performance polymeric materials is able to improve the membrane separation performance, but such an approach is always accompanied with unexpected risks, economic costs and development duration [14]. Thus, a more feasible approach is highly required. Some of the strategies that have been employed to overcome this challenge are improving the existing membrane performance via polymer blends [15] or the incorporation of inorganic fillers (e.g., graphene oxide (GO), zeolite, and silica) [16,17,18,19,20], modifying the membrane selective layer via surface coating [21,22,23,24] and developing a unique dual-layer membrane structure [25,26,27].

Of these strategies, surface dip-coating is commonly adopted by the researchers as one of the most practical methods to improve membrane performance. This method is easy to perform and has a high chance of producing membranes with enhanced properties owing to the synergistic effects resulting from the use of different materials. The coating method was started in the 1980s, when Henis and Tripodi [28] came out with a solution to overcome the defects at the top selective layer of hollow fiber membrane by coating a silicone rubber layer to stop the leak through the defects. Their technique was later adopted by Monsanto, leading to the Prism membrane for the separation of hydrogen from other gases [29].

Compared to single-layer-coated membranes [30,31], studies have shown that forming a multilayer on the membrane surface is more advantageous as it combines the positive features of two different coating materials [32,33,34]. A multilayer-coated membrane is generally composed of three different layers, with each layer playing a different role. The first layer is the thick porous support membrane, which offers mechanical strength. It is followed by a gutter layer (first coating layer) that can reduce the penetration of the selective layer (second coating layer) into the membrane pores. Ultimately, it is the top selective layer that governs the gas transport mechanism [33].

The properties of the selective layer are reported to be further improved upon the incorporation of inorganic nanomaterials [34,35]. Zulhairun et al. [35], for instance, introduced a metal organic framework (MOF)-Cu_3_(BTC)_2_ into a polydimethysiloxane (PDMS) coating solution and reported that the hybrid coating layer improved the CO_2_ permeance of the PDMS-coated membrane (without Cu_3_(BTC)_2_ incorporation) by 28.6%, leading to an increase in the CO_2_/CH_4_ and CO_2_/N_2_ selectivities from 28.06 to 30.46 and from 31.34 to 33.40, respectively. The improved membrane performance was explained by Cu_3_(BTC)_2_′s higher affinity towards CO_2_ due to the coordinatively unsaturated copper sites in its crystal network. This provided an exceptionally high adsorptive capability for polar molecules, resulting in an increase in the CO_2_ permeance and selectivities.

Liang et al. [34] also reported that when a beta-cyclodextrin (β-CD)/intrinsic microporosity (PIMs) blend solution was applied to the PDMS-coated membrane surface, the resultant multilayer-coated membrane was able to increase the N_2_, O_2_, and CO_2_ permeance of the membrane (without β-CD incorporation) by 53.4%, 63.9%, and 66.8%, respectively. In addition, the presence of β-CD in the PIMs layer was also reported to enhance the O_2_/N_2_ and CO_2_/N_2_ selectivities by 9.4% and 8.8%, respectively. The authors elucidated that the incorporation of three-dimensional β-CD into the main polymer chains of PIM created more micro-pore volumes or fractional free volumes (FFV), which increased the CO_2_ sorption capacity.

In view of this, the main objective of this work is to develop a multilayer-coated hollow fiber membrane by incorporating GO nanosheets into the selective layer of the membrane. A GO nanosheet was selected in this work, as it has been previously demonstrated to improve the gas separation performance of mixed matrix membranes (MMM) [16,17,36,37]. By introducing the GO into the selective layer, we can minimize the amount of the GO used for membrane modification. In this work, the multilayer-coated membrane was developed by forming a PDMS gutter layer on the surface of the PSF membrane, followed by coating the Pebax layer with different amounts of GO (ranging from zero to 1.0 wt%). The effects of GO loading on the membrane surface properties were then investigated using a series of analytical instruments before its performance was assessed for CO_2_/CH_4_ and O_2_/N_2_ separation.

## 2. Materials and Methods

### 2.1. Materials

Polysulfone (PSF, Udel^®^ P-3500)—the forming material for hollow fiber membrane—was procured from Solvay. N,N-dimethylacetamide (DMAc) and ethanol obtained from Merck and tetrahydrofuran (THF) obtained from QReC were used as co-solvents for the dope solution preparation. GO nanosheets were self-synthesized in this work using graphite powder supplied by Sigma Aldrich. Other chemicals used to synthesize GO were sulfuric acid (H_2_SO_4_) (95–97%, Merck, Kenilworth, NJ, USA), sodium nitrate (NaNO_3_) (99.5%, Riedel-de Haen, NJ, USA), potassium permanganate (KMnO_4_) (99%, Sigma-Aldrich, St. Louis, MO, USA), and hydrogen peroxide (H_2_O_2_) (30%, Merck). For the process of GO washing, barium chloride 2-hydrate (BaCl_2_(H_2_O)_2_) (99%, Riedel-de Haen), hydrochloric acid (HCl) (37%, Merck), and acetone (>99%, Merck) were used. To prepare a selective coating layer on the outer surface of hollow fiber membrane, Pebax^®^ MH 1657 in ethanol obtained from Arkema was used. For the gutter layer formation, PDMS coating solution was prepared using a Sylgard^®^ 184 Silicone Elastomer base and curing agents supplied by Dow Corning. To evaluate the membrane separation performance, four purified gases—i.e., CO_2_, CH_4_, O_2_, and N_2_—supplied by Mega Mount Industrial Gases Sdn. Bhd., Malaysia, were used.

### 2.2. PSF Hollow Fiber Membrane Fabrication

The hollow fiber spinning solution consisted of 25 wt% PSF, 32.5 wt% DMAc (non-volatile solvent), 32.5 wt% THF (volatile solvent), and 10 wt% ethanol. To prepare the spinning dope, DMAc and THF were first mixed, followed by adding PSF slowly to the mixture to avoid polymer agglomeration. Then, ethanol was added and the solution was continuously stirred for 24 h at 60 °C to make the solution completely uniform. Lastly, the solution was cooled down to room temperature and degassed to remove any micro-bubbles prior to its use for hollow fiber spinning by a self-customized spinning machine via the dry-jet-wet-quench spinning method. The details of the spinning conditions were reported in our earlier publication [38,39].

Before starting the spinning process, the dope solution was transferred from a Schott bottle into a stainless-steel feed reservoir. A gear pump was used to deliver the solution at an extrusion rate of 1 mL/min from the reservoir to a spinneret with outer diameter (OD)/inner diameter (ID) dimension of 0.60 mm/0.30 mm. The bore fluid, composed of 90 vol% NMP and 10 vol% pure water, was channeled simultaneously into the spinneret using a syringe pump at a bore fluid flow rate of 0.67 mL/min. After extrusion, the pristine hollow fiber was guided manually into a coagulation water bath, which was located 4 cm below the spinneret. In the coagulation bath, the fiber thread was guided through several rollers immersed in the water until it was collected at a wind-up drum (with 50% of its area immersed in water) at a take-up speed of 12 m/min. Once the spinning process was completed, the hollow fibers were taken off from the drum and transferred to another water bath, in which the hollow fibers were stored for at least 2 days to remove excess solvents. The hollow fibers were then immersed in pure methanol for 4 h for solvent exchange, before being dried at room condition for at least 2 days before it was used for module preparation.

### 2.3. GO Synthesis and Characterization

The synthesis of GO nanosheets was carried out in accordance with Hummers′ method. First, 3 g of graphite and 1.5 g of NaNO_3_ were added to a concentrated H_2_SO_4_ solution (69 mL). The mixture was then cooled down immediately to 0 °C using an ice bath, followed by continuous stirring for 15 min. Then, 9 g of KMnO_4_ was added slowly into the mixture while maintaining the temperature below 20 °C to avoid overheating. After stirring for 2 h, the ice bath was replaced by a water bath and the mixture was further stirred for 30 min at 35 °C. About 150 mL of RO water was then added into the mixture, followed by 15 min of stirring. During the process, the mixture temperature was kept below 95 °C. Finally, the reaction was terminated by adding 500 mL of RO water and 15 mL of 30% H_2_O_2_ solution in order to reduce the residual permanganate and manganese dioxide to colorless soluble manganese sulfate.

The resultant mixture was then filtered, followed by washing using 5% HCl solution until sulfate could not be detected by BaCl_2_. Afterwards, the resultant GO was washed by RO water and centrifuged until the supernatant reached pH 5–6. The GO in the aqueous solution was then ultrasonicated for 1 h, followed by 2 h centrifugation at 4000 rpm to remove large and not fully exfoliated GO. The treated GO was then dried in a vacuum oven for 12 h at 60 °C. To further purify the GO, the dried GO was dissolved in a large amount of acetone and stirred vigorously. At last, the GO solution was filtered and the obtained GO cake was dried in a vacuum oven at 60 °C for 6 h to produce highly purified GO.

The crystallinity of the self-developed GO was characterized by X-ray diffraction analysis (XRD) to obtain the spectrum information. An X-ray diffractometer with Cu Kα radiation (λ = 0.154 nm, D/max-RB 12 kW Rigaku) was used and operated at 30 mA and 40 kV from 5° and 80° with a step increment rate of 0.05°/min. In addition, the structural morphology of GO was observed at 120 kV using a transmission electron microscope (TEM) (HT7700, Hitachi, Chiyoda City, Tokyo, Japan). Prior to the TEM analysis, a small amount of the GO sample was prepared by dispersing it in ethanol, followed by 30 min of ultrasonication before the GO solution was placed onto a copper grid and dried at room conditions for 3 days.

### 2.4. Preparation of Composite Hollow Fiber Membrane

#### 2.4.1. Coating Solution Preparation

Prior to the multilayer-coated membrane fabrication, two types of coating solutions were firstly prepared. First, a 3 wt% PDMS solution was prepared to form a gutter layer on the PSF hollow fiber membrane surface. This solution was prepared by mixing a predetermined amount of elastomer (5.46 g) in n-hexane (194 g) under magnetic stirring for 15 min. Then, weighed hardener (0.54 g) was added and the mixture was further stirred for 30 min to achieve complete mixing. Heating was not applied during the PDMS solution preparation, and the prepared solution could be used immediately after it was produced. Separately, a selective layer solution was prepared by adding 3 wt% Pebax into the ethanol/water mixture with a mass ratio of 70/30. The completely dried Pebax pellets were added to the solvent mixture, followed by overnight stirring at room temperature. Afterwards, the temperature was raised to 80–85 °C, while the mixture was kept stirring in order to ensure the swollen Pebax pellets were completely dissolved. The prepared solution was then cooled down to room temperature before a specific amount of GO (i.e., 0, 0.2, 0.4, 0.6, 0.8, and 1.0 wt%) was added. The solution was then stirred using a magnetic stirrer for 1 day, followed by ultrasonication for 30 min to form a homogenous solution. During the ultrasonication process, the temperature of the water bath was maintained below 35 °C. The amount of GO added into Pebax solution was calculated using the following equation:

(1) $$\mathrm{GO}\;\mathrm{Loading}\;\left(\%\right)=\;\frac{\mathrm{Mass}\;\mathrm{GO}\;\left(g\right)\;}{\mathrm{Mass}\;\mathrm{GO}\;\left(g\right)+\mathrm{Mass}\;\mathrm{Pebax}\;\left(g\right)}\times \;100$$

#### 2.4.2. Multilayer Hybrid Coating Procedure

**Figure 1:**
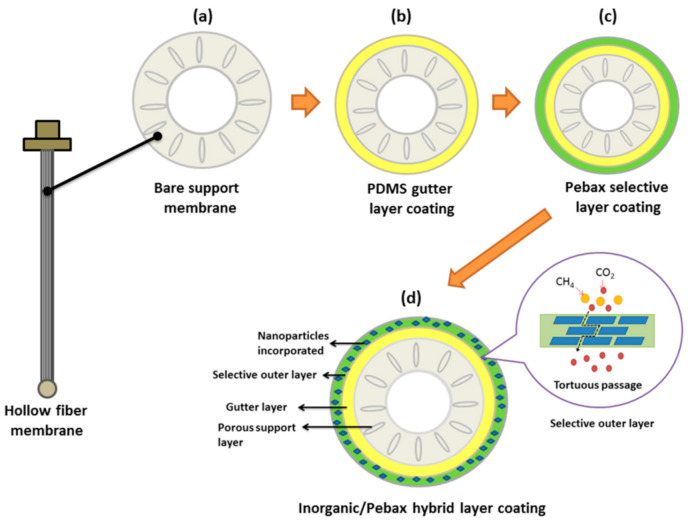
Illustration of the hybrid coating approach for the fabrication of multilayered hollow fiber membrane (potted in a module), (**a**) uncoated membrane, (**b**) PDMS-coated membrane, (**c**) Pebax-coated membrane, and (**d**) multilayer hybrid-coated membrane.

The hybrid coating process adopted in this work was based on the dip-coating method. At first, hollow fibers (length: ~10 cm; OD: ~0.04 cm; total effective surface area: ~1.23 cm^2^) were potted in a stainless-steel adaptor. Only one side of hollow fiber lumen was opened while another end was completely sealed by bi-component epoxy resin (E-30CL™ Hysol^®^ Epoxy, Loctite^®^). The prepared membrane adaptor was then immersed in 3 wt% PDMS solution for 10 min to form a gutter layer. Afterwards, the PDMS-coated membrane was further coated with a secondary layer by immersing it in the Pebax solution containing different amounts of GO (up to 1.0 wt%). For each surface coating, 2 days were required to achieve complete drying. Figure 1 illustrates the coating procedure employed in this work for the fabrication of a multilayered hollow fiber membrane.

### 2.5. Membrane Evaluation and Characterization

Prior to the membrane performance evaluation, the hollow fiber membrane potted in an adaptor was first placed within a stainless-steel housing. As the selective layer was established on the outer layer of hollow fiber membrane, the separation test was carried out in the outside-in filtration mode—i.e., gas was fed onto the outer layer of membrane while permeated gas was collected from the lumen side of the fiber. The performances of the membranes with respect to the gas permeance and gas pair selectivity were evaluated using a self-customized gas permeation system, as reported in our previous work [39]. In the present work, four gases (O_2_, N_2_, CH_4_, and CO_2_) were used and the sequence of gas permeation was started with “slow” gas (CH_4_ and N_2_) followed by “fast” gas (CO_2_ and O_2_). Such an approach is used to minimize the impacts caused by “fast” gas, which could swell the space between the polymer chains and disrupt the polymer structure, affecting the separation process of gas molecules with bigger sizes [40]. Furthermore, an acidic gas such as CO_2_ has to be avoided in the first place, as it could easily change the membrane morphological structure, causing a clogging problem [41]. The membrane separation performance was studied at 5 bar at standard room temperature (25 ºC). The permeated gas collected from the membrane was measured based on the soap-bubble flow method. To determine the gas permeance, (*P_i_*/*l*), the following equation was used:

(2) $$\frac{P_{i}}{l}=\;\frac{Q_{i}}{A\Delta P}\frac{273.15\;\times 10^{6}}{T},$$

where (*P_i_*/*l*) is the gas permeance of a membrane in GPU (1 GPU = 1 × 10^−6^ cm^3^ (STP)/cm^2^ s cmHg), *i* represents the penetrating gas *i*, *Q_i_* is the volumetric flow rate of gas (cm^3^/s) permeated through the membrane at a temperature *T* and 1 atm, Δ*P* is the trans-membrane pressure difference (cmHg), *T* is the temperature (K) of the ambient environment in which the permeation experiment is performed, and *A* is the effective membrane surface area (cm^2^). The ideal gas pair selectivity, $\alpha_{i/j}$, meanwhile was determined by the permeance ratio of the gas component *i* over *j*:

(3) $$\alpha_{i/j}\;=\;\frac{\left(P_{i}/\;l\right)}{(P_{j}/l)}.$$

In this work, only the gas pair selectivities of CO_2_/CH_4_ and O_2_/N_2_ were reported. In addition, the effect of feed pressure (ranging from 1 to 9 bar, T: 25 ºC) on the membrane performance as well as membrane stability for up to 50 h of operation (at fixed pressure) was also investigated to compare the performance of the best performing multilayer-coated membrane with that of the control membrane. Due to the limitation of testing facilities, the stability test was run only for 10 h per day and was continued in the following days for a complete 50 h period.

Several instruments were used to characterize the physiochemical properties of the developed membranes. FTIR/ATR instrument (Thermo Scientific Nicolet, iS10, NY, USA) was used to analyze the functional groups present in the coating layer. The spectrum ranges were set at 4000–400 cm^−1^. Since the penetration depth of FTIR was not more than a few microns, such an analysis could represent the top coating layer. In order to confirm the presence of GO in the coating layer, an X-ray photoelectron spectroscopy (XPS) analysis was performed using ESCALAB 250 (Thermo Fisher Scientific, Waltham, MA, USA). The spectra were recorded using monochromatized Al Kα radiation (1486.6 eV). The outer surface and cross-sectional morphologies of the hollow fiber membranes were examined using a field emission scanning electron microscope (FESEM) (SU8000, Hitachi). Prior to the FESEM analysis, one fiber was randomly chosen and fractured in liquid nitrogen to obtain a perfect-cutting edge before it was attached to the sample stub for examination. Both the cross-section and outer surface of the membranes were then gold sputtered to avoid over-charging during characterization. The membrane surface roughness was also examined using an atomic force microscope (AFM) (Park Systems NX10) in non-contact mode (cantilever: NANOSENSORS^™^ PPP-NCHR).

## 3. Results and Discussion

### 3.1. Characterization of GO

**Figure 2:**
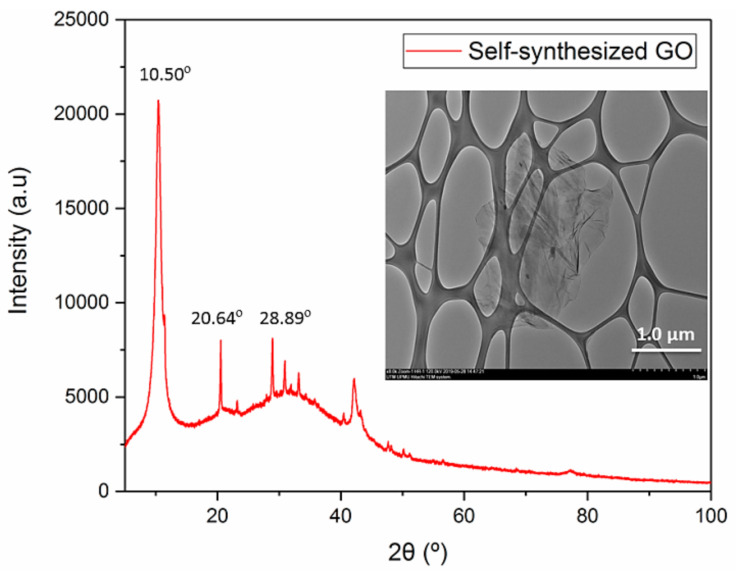
XRD spectrum of self-synthesized GO nanosheet (Inset: TEM image of GO nanosheet).

The purity and morphology of the self-synthesized GO are confirmed based on the results shown in Figure 2. The presence of a strong diffraction peak at 2θ of 10.50° indicates the successful formation of GO. It suggests the exfoliation of GO due to the attachment of functional groups during the oxidation process [42,43,44]. The sharp peak is strong evidence of the crystal plane of GO with a spacing of 8.46 Å [45]. Another two diffraction peaks at 20.64° and 28.89° are attributed to unreacted graphite flakes and manganese ions [42]. The TEM image further shows that the synthesized GO is in single flake form, with a lateral size of several microns. Our findings are in good agreement with other published works [42,44]. With respect to the thickness, the GO was found in the range of 0.9–1.0 nm, as reported in our previous study [42].

### 3.2. Effect of GO Loadings on the Membrane Properties

**Figure 3:**
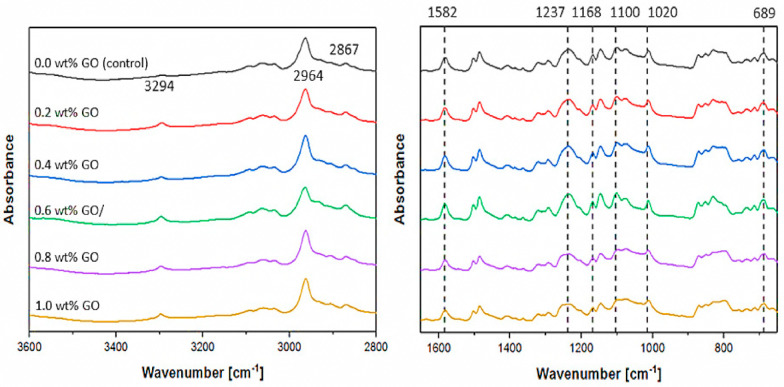
FTIR spectra of multilayer-coated membranes with different GO loadings.

Figure 3 compares the FTIR spectra of the multilayer-coated membranes made of different GO loadings from zero to 1.0 wt%. The results show that a low intensity peak at 3294 cm^−1^ appeared for all GO-containing membranes, while it was not for the control membrane (without GO). This could be due to the O−H functional groups of GO. The presence of the top Pebax layer could be further verified by the peaks at 2867, 1582, and 1168 cm^−1^, which correspond to the –CH_3_ stretching, N–H, and C–O ester bond of the Pebax layer, respectively [46]. The PDMS gutter layer, meanwhile, could be detected by the peaks that appeared at 1100, 1020, and 689 cm^−1^. They are attributed to Si–CH_2_ bonds, Si–O–Si, and Si–CH_3_ stretching, respectively [47]. On the other hand, the presence of peaks at 2964 and 1237 cm^−1^, as detected on the surface of all studied membranes, was due to the CH_2_ aliphatic group and O=S=O stretching vibration contributed by the PSF that was used to fabricate the hollow fiber membrane [48].

**Figure 4:**
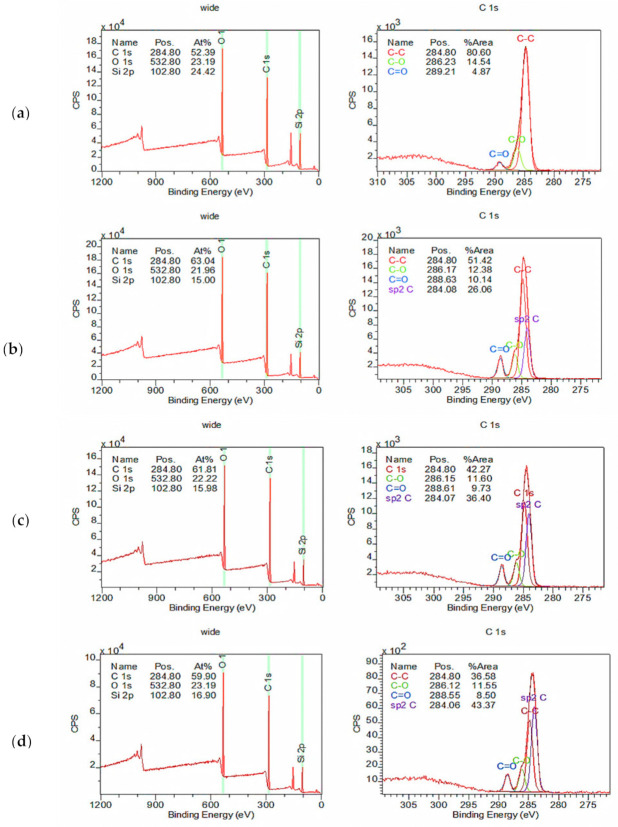
XPS spectra of a complete survey spectrum (left) and C1s spectrum (right) of membrane with different nanomaterial loadings: (**a**) 0.0 wt%, (**b**) 0.4 wt%, (**c**) 0.8 wt%, and (**d**) 1.0 wt% GO.

Figure 4 shows the XPS surface analysis of the membranes containing different amounts of GO. Four elements—i.e., C, N, O, and Si—are supposed to be included in this analysis, but the signal from the N element (at ~400 eV) in Pebax could not be detected possibly due to its extremely low intensity compared to that of other elements. The coating layer of Pebax is significantly thinner compared to the PDMS gutter layer, which can be confirmed based on the presence of Si element. The existence of C and O element, meanwhile, can be attributed to the GO embedded and/or Pebax coating layer. Further analysis based on the high-resolution spectrum of C1s strongly suggests the existence of GO in the developed membranes. The peak of the *sp2* hybridization state of carbon (~284 eV) is only found in the membranes containing GO, and its intensity increases with the increasing GO loading. The presence of *sp2* of carbon is attributed to the characteristics of GO, as reported elsewhere [45].

**Figure 5:**
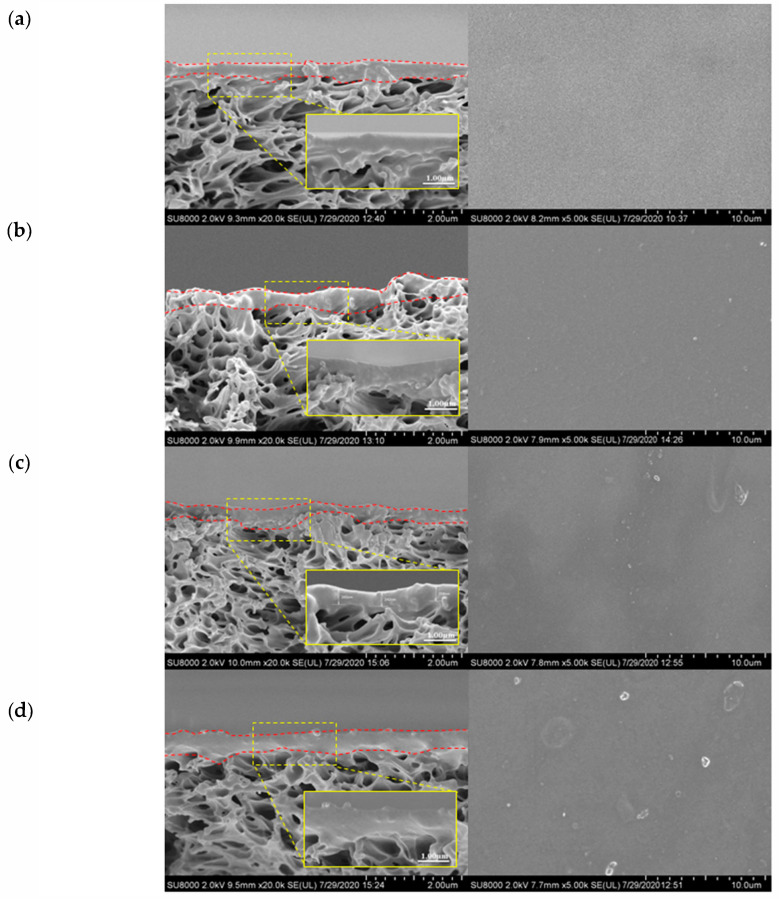
FESEM images of cross-section (left, inset: enlarged image of the coating layer) and surface (right) of the membranes with different GO loadings: (**a**) 0.0 wt%, (**b**) 0.4 wt%, (**c**) 0.8 wt%, and (**d**) 1.0 wt% GO.

Figure 5 shows the FESEM cross-sectional and surface images of the membranes with and without GO incorporation. It can be seen clearly from the membrane cross-section images that a dense coating layer is successfully formed atop of the membrane. Nevertheless, there is not much difference among these membranes, except for the membrane with the highest GO loading (1.0 wt%) that exhibits a slightly rougher surface. The coating layer thickness is almost the same (~0.30 µm) regardless of the GO content, since the concentrations of PDMS and Pebax in the coating dopes were the same for all the membranes, except for the amount of GO added. With respect to the membrane surface morphology, it can be clearly seen that the membranes with GO exhibit a rougher surface compared to the control membrane (without GO), and it becomes rougher with the increasing GO loading. This can be mainly due to the wrinkly structure of GO itself and possible particle aggregation, particularly in the case where the GO loading was the highest.

**Figure 6:**
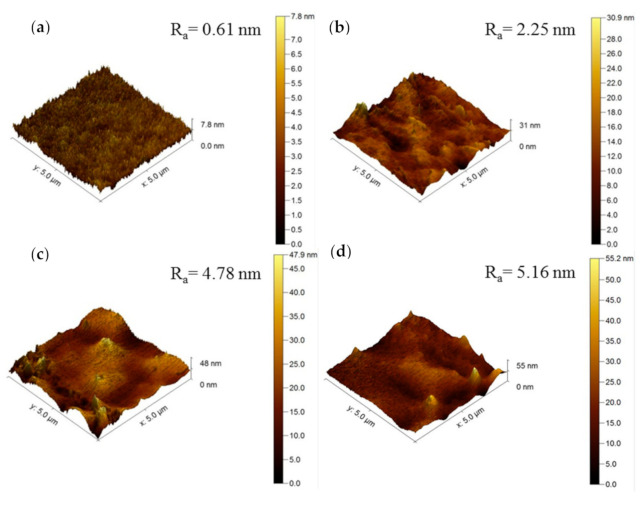
3D AFM images of membranes with different nanomaterial loadings: (**a**) 0.0 wt%, (**b**) 0.4 wt%, (**c**) 0.8 wt%, and (**d**) 1.0 wt% GO.

The increase in the membrane surface roughness upon GO incorporation is further supported by the AFM images, as shown in Figure 6. The roughness value (R_a_) of the membrane is gradually increased from 0.61 nm in the control membrane (without GO) to 2.25, 4.78, and 5.16 nm in the membranes containing 0.4, 0.8, and 1.0 wt% GO, respectively. The increase in surface roughness is expected due to incompatibility between the nanomaterial and the polymer matrix. However, it must be pointed out that the change in the surface roughness in fact is very small considering that the value is reported in nm scale.

### 3.3. Impacts of GO Loadings on Membrane Performance

The impacts of Pebax concentration (1–9 wt%) on the gas separation performance of the multilayer-coated membranes for gas separation were recently studied by our group in the absence of GO [39]. Although optimizing the properties of the Pebax selective layer atop PDMS gutter layer could improve the membrane gas separation performance, the introduction of a small quantity of GO into the Pebax layer is expected to further improve the selectivity due to the formation of tortuous paths within the outer selective layer.

**Figure 7:**
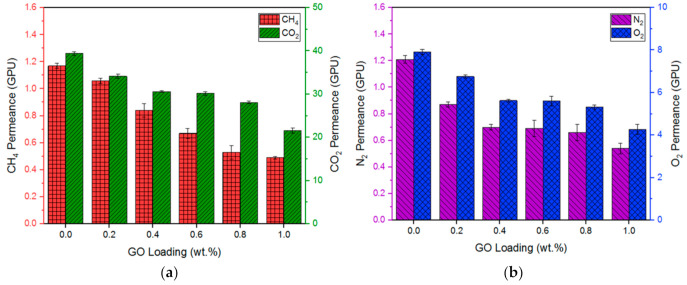
Pure gas permeance of multilayer-coated membranes with different GO loadings at 5 bar: (**a**) CH_4_ and CO_2_ and (**b**) N_2_ and O_2_.

Figure 7 shows the pure gas permeance of the multilayer-coated membranes with different GO loadings at 5 bar. As can be seen, the gas permeance of membrane is decreased upon the incorporation of 0.2 wt% GO. For instance, the CO_2_ and O_2_ permeance is decreased from 39.40 GPU to 34.15 GPU and 7.91 GPU to 6.76 GPU, respectively, from zero to 0.2 wt% GO loading. The gas permeance continues to decrease with increasing GO loading from 0.2 wt% to 1.0 wt%, but at a small degree of decline. This decrease in gas permeance could be due to the formation of the tortuous path within the Pebax selective layer, as mentioned above. The increased tortuous path eventually leads to an increasing membrane transport resistance, making gas molecules more difficult to diffuse [16]. Attempt was also made in this work to prepare a multilayer coated membrane with 1.2 wt% GO. However, no gas separation test was able to be conducted using this membrane, as the membrane was burst during the test. The presence of a large amount of GO in the selective layer creates excessively tortuous paths, which makes it almost impossible for gas molecules to pass through it.

**Figure 8:**
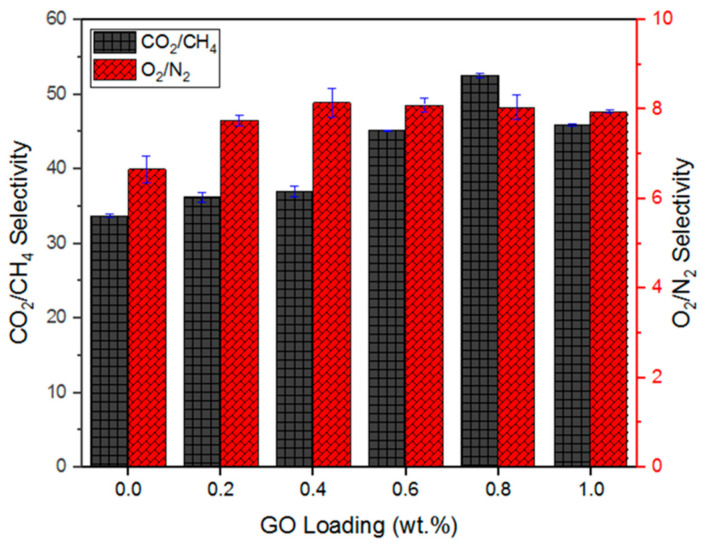
Gas pair selectivity performance of the GO/Pebax-hybrid coated membranes.

The CO_2_/CH_4_ and CO_2_/N_2_ selectivities are compared to the control membrane, as shown in Figure 8. With respect to the membrane with 0.2 wt% GO, its CO_2_/CH_4_ and O_2_/N_2_ selectivities were increased by 6.1% and 19.1%, respectively, from the control membrane, recording the values of 36.36 and 7.92 GPU. The gas pair selectivities continued to improve until they reached maxima at 0.8 wt% GO. At 0.8 wt% GO, the membrane showed CO_2_/CH_4_ and O_2_/N_2_ selectivities of 52.57 and 8.05, respectively. These values are 56.1% and 20.9% higher than those of the control membrane. However, the CO_2_/CH_4_ and O_2_/N_2_ selectivities decreased by 12.6% and 1.2%, respectively, from 0.8 wt% to 1.0 wt% GO loading. This can be attributed to the formation of the narrower tortuous path formed, which causes higher permeation resistances even for smaller gas molecules (e.g., CO_2_ and O_2_), resulting in a decrease in the gas pair selectivities.

When GO is incorporated in the Pebax coating layer, it tends to increase the length of the tortuous path within the selective layer, and this makes the membrane more selective to gases based on gas molecular size, i.e., CH_4_ (0.38 nm) > N_2_ (0.36 nm) > O_2_ (0.35 nm) > CO_2_ (0.33 nm). The increase in the tortuous path length would create a higher resistance to bigger gas molecules (i.e., CH_4_ and N_2_) compared to smaller gas molecules (CO_2_ and O_2_), leading to increased gas pair selectivity [16]. It can be also seen that the selectivity of CO_2_/CH_4_ is much higher than that of O_2_/N_2_ selectivity, and this is due to the greater molecular size difference between CH_4_ and CO_2_ compared to N_2_ and O_2_.

Table 1 compares the performance of the best performing membrane in this work with the other membranes reported in the literature [34,35,49,50,51,52,53,54,55]. It can be seen that the performance of our multilayer-coated membrane with 0.8 wt% GO loading is comparable with or better than the other works in terms of CO_2_/CH_4_, O_2_/N_2_, and CO_2_/N_2_ separation.

### 3.4. Effect of Feed Pressure on Multilayer-Coated Membranes

**Figure 9:**
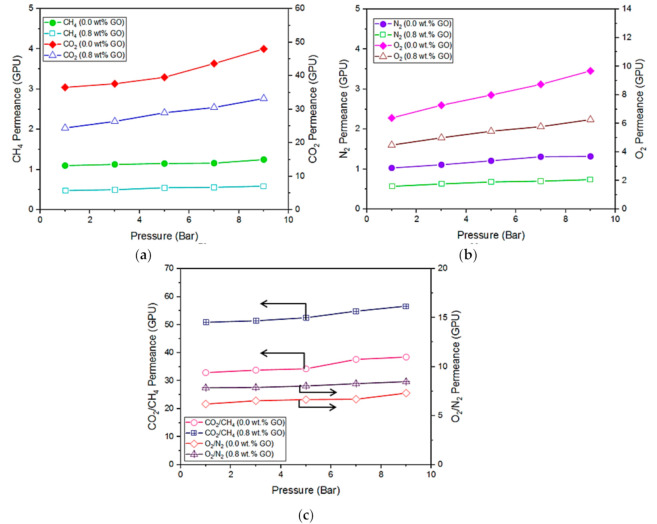
Effect of feed pressure on (**a**) CO_2_, CH_4_, and (**b**) O_2_, N_2_ gas permeance of 0.0 wt% and 0.8 wt% GO and their respective gas pair selectivity (**c**).

The permeability of polymeric membranes tends to increase with an increasing operating pressure; hence, plasticization is a key issue for the industrial use of polymeric membranes. The effects of operating pressure on the gas separation performance of the multilayer coated membranes are presented in Figure 9. The pure gas permeabilities of the control membrane (without GO incorporation) are found to increase more than the 0.8 wt% GO-loaded membrane when the pressure is increased from 1 to 9 bar. In particular, the CO_2_ permeance of the control membrane is significantly increased at the operating pressure above 5 bar. Such an increasing trend is the indication of membrane plasticization caused by the high-pressure CO_2_ where the increased permeability is due to increased diffusivity of the gas through the membrane, rather than increased solubility [56]. At a critical pressure (>5 bar), the CO_2_ permeability begins to significantly increase with pressure. The minimum in the permeability isotherm is known as the plasticization pressure and is used to qualitatively describe the maximum CO_2_ pressure at which glassy polymeric membranes can operate.

The plasticization of the GO-incorporating membrane was less than that of the control membrane in the above pressure range. The results clearly suggest the positive role of GO in improving the anti-plasticization ability of the multilayer-coated membrane by reducing the chain mobility of Pebax. With respect to the CH_4_ and N_2_ permeation, no obvious plasticization effect is found on both membranes.

With respect to gas pair selectivity, the membrane with GO incorporation always exhibits higher gas pair selectivities for both CO_2_/CH_4_ and O_2_/N_2_ than the control membrane in the entire pressure range studied. Increasing the operating pressure is found to have greater impact on the CO_2_/CH_4_ separation compared to the O_2_/N_2_ separation. The CO_2_/CH_4_ selectivity of the membrane with GO incorporation was increased by 11.4% by increasing the pressure from 1 to 9 bar, while the O_2_/N_2_ selectivity was increased marginally (8.5%). However, it must be pointed out that the selectivities would be affected when a mixed gas is used. For instance, the permeance of CH_4_ would be increased in the presence of CO_2_ during mixed gas testing, and this lowers the CO_2_/CH_4_ selectivity of the test that uses a single gas for testing.

### 3.5. Performance Stability of Multilayer Membranes

**Figure 10:**
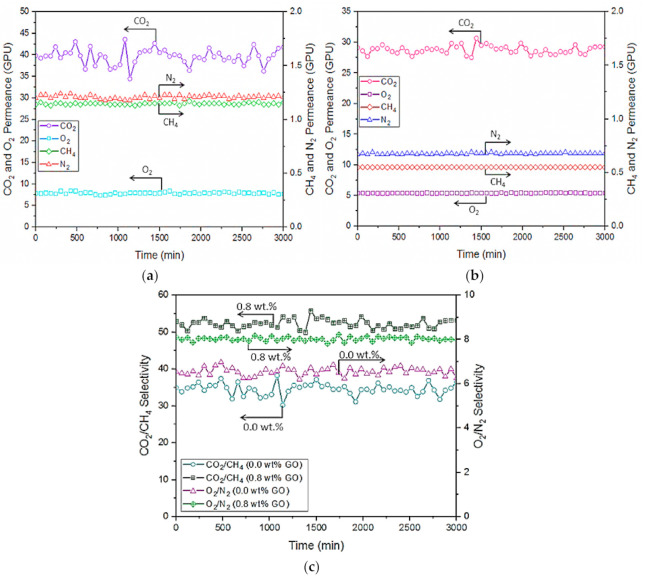
Long-term performance stability of multilayer-coated membrane without GO (**a**) and with 0.8 wt% GO (**b**) and their respective gas pair selectivity (**c**).

Performance stability is one of the main concerns for the membrane to be practically used for industrial applications. Figure 10 shows the stability profile of multilayer-coated membrane for CO_2_/CH_4_ and O_2_/N_2_ separation at 5 bar for up to 3000 min. It is found that both membranes exhibit a very stable performance for CH_4_, O_2_, and N_2_ permeation over time. However, both membranes experienced fluctuation in the CO_2_ permeance. Dai et al. [16] also reported the greater fluctuation of CO_2_ permeance compared to N_2_ permeance in their study that used imidazole functionalized GO/Pebax mixed-matrix membranes. In our work, the degree of fluctuation in the control membrane was much higher than that of the membrane with GO incorporated owing to its relatively lower gas permeance. Our findings reveal that the presence of GO in the Pebax selective layer of membrane could improve the membrane performance stability while showing a higher gas pair selectivity for CO_2_/CH_4_ and CO_2_/N_2_.

## 4. Conclusions

In this work, we investigated the impacts of GO nanosheets on the properties of the multilayer-coated hollow fiber membranes for CO_2_/CH_4_ and O_2_/N_2_ separation. Different quantities of GO (0 to 1.0 wt%) were loaded into the Pebax selective layer of multilayer-coated membranes, and the effects on the membrane surface physiochemical properties were investigated using a series of analytical instruments, followed by performance evaluation. The XPS results clearly showed the existence of GO in the membrane selective layer, and the greater the amount of GO added, the higher the peak intensity detected at ~284 eV. This is due to the *sp2* hybridization state of carbon resulting from GO nanosheets. In terms of the coating layer morphology, increasing the GO amount only affected the membrane surface roughness without altering the entire coating layer thickness. Our results also indicated that the addition of 0.8 wt% GO in the Pebax coating layer could produce the best performing multilayer-coated membrane, showing 56.1% and 20.9% enhancement in the CO_2_/CH_4_ and O_2_/N_2_ gas pair selectivities, respectively, in comparison to the membrane without GO incorporation. The improvement is due to the increase in the tortuous path length in the selective layer upon GO incorporation, which created a higher resistance to the larger gas molecules (CH_4_ and N_2_) compared to the smaller gas molecules (CO_2_ and O_2_). Furthermore, the best performing membrane also demonstrated a lower degree of plasticization compared to the control membrane. It showed a very stable performance for long-term operation (up to 50 h) by recording the CO_2_/CH_4_ and O_2_/N_2_ gas pair selectivities of 52.57 and 8.05, respectively.

## Figures and Tables

**Table 1:** Comparison of membranes with a hybrid coating layer for gas separation.

Support Polymer	^1^ Hybrid Coating Material	Configuration	Pure Gas Permeance (GPU)				O_2_/N_2_	CO_2_/CH_4_	CO_2_/N_2_	Reference
			O_2_	N_2_	CO_2_	CH_4_				
PAN	β-CD/PIM/PDMS	Hollow fiber	^2^ 69.0	^2^ 21.50	^2^ 483.40	-	3.20	-	22.50	[34]
PSF	Cu_3_(BTC)_2_/PDMS	Hollow fiber	-	3.10	109.20	3.70	-	29.51	35.23	[35]
PSF	TEOS/PDMS	Flat sheet	-	-	21.50	3.90	-	39.81	-	[49]
PSF	Fe(DA)/Pebax	Hollow fiber	-	1.61	90.00	-	-	56.00	-	[50]
PAN	ZIF-7/Pebax	Flat sheet	-	0.37	39.00	0.89	-	44.00	105.00	[51]
PEI	ZIF-8/Ultem	Hollow fiber	-	1.21	34.00	-	-	-	28.00	[52]
PVDF	ZIF-8/Pebax	Hollow fiber	-	10.94	350.00	25.00	-	14.00	32.00	[53]
PVDF	GO/Pebax	Hollow fiber	-	9.65	415.00	-	-	-	43.00	[54]
PPO	GO/SHPAA/PVA	Hollow fiber		26.61	825.00	41.25	-	20.00	31.00	[55]
PSF	GO/Pebax	Hollow fiber	5.32	0.66	28.08	0.53	8.05	52.57	42.55	This study

^1^ β-CD: beta-cyclodextrin; Cu_3_(BTC)_2_: copper benzene-1,3,5-tricarboxylate; TEOS: tetraethyl orthosilicate; Fe(DA): iron dopamine; ZIF: zeolitic imidazolate framework; GO: graphene oxide. ^2^ The permeance was measured in Barrer.
